# Crusted Demodicosis in an Immunocompetent Pediatric Patient

**DOI:** 10.1155/2014/458046

**Published:** 2014-10-12

**Authors:** Guillermo Antonio Guerrero-González, Maira Elizabeth Herz-Ruelas, Minerva Gómez-Flores, Jorge Ocampo-Candiani

**Affiliations:** Dermatology Department, Hospital Universitario “Dr. José Eleuterio González,” Universidad Autónoma de Nuevo León, Avenida Francisco I. Madero Poniente s/n y Avenida Gonzalitos, Colonia Mitras Centro, 64460 Monterrey, NL, Mexico

## Abstract

Demodicosis refers to the infestation by *Demodex* spp., a saprophytic mite of the pilosebaceous unit. Demodex proliferation can result in a number of cutaneous disorders including pustular folliculitis, pityriasis folliculorum, papulopustular, and granulomatous rosacea, among others. We report the case of a 7-year-old female presenting with pruritic grayish crusted lesions over her nose and cheeks, along with facial erythema, papules, and pustules. The father referred chronic use of topical steroids. A potassium hydroxide mount of a pustule scraping revealed several *D. folliculorum* mites. Oral ivermectin (200 *μ*g/kg, single dose) plus topical permethrin 5% lotion applied for 3 consecutive nights were administered. Oral ivermectin was repeated every week and oral erythromycin plus topical metronidazole cream was added. The facial lesions greatly improved within the following 3 months. While infestation of the pilosebaceous unit by *Demodex folliculorum* mites is common, only few individuals present symptoms. Demodicosis can present as pruritic papules, pustules, plaques, and granulomatous facial lesions. To our knowledge, this is the first reported case of facial crusted demodicosis in an immunocompetent child. The development of symptoms in this patient could be secondary to local immunosuppression caused by the chronic use of topical steroids.

## 1. Introduction

Demodicosis refers to the infestation by* Demodex* spp., a saprophytic mite of the pilosebaceous unit. Colonization usually occurs starting adolescence or afterwards, when sebaceous glands mature and multiply. A high prevalence of Demodex (80–100%) by the age of 50 years is proposed [1], although few individuals develop symptoms. Demodex proliferation can result in a number of cutaneous disorders including pustular folliculitis, pityriasis folliculorum, and papulopustular and granulomatous rosacea [2]. Several factors may be implicated in the development of pathogenic forms, including increased density of the mite, immune system disorders such as HIV infection, and the use of corticosteroids.

## 2. Case Presentation

**Figure 1:**
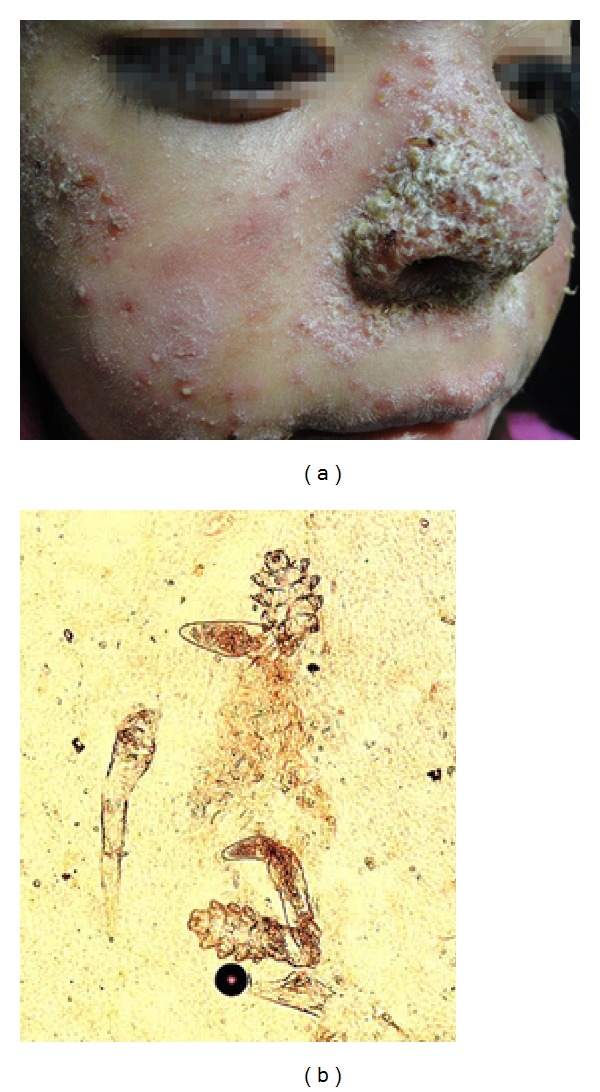
(a) Facial erythema, grayish crusted lesions, papules, and pustules. (b) Skin scraping revealing* D. folliculorum* mites.

**Figure 2:**
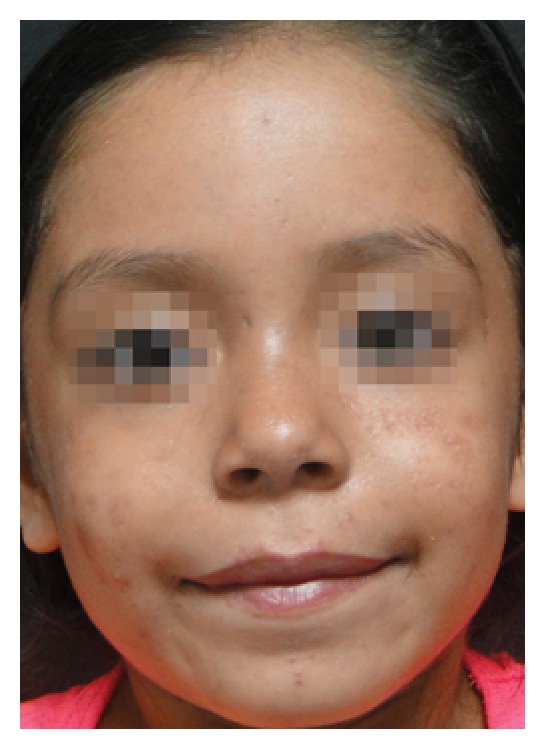
Clinical resolution after 3 months of treatment.

We report the case of a 7-year-old female patient presenting with pruritic grayish and yellowish crusted, scaly plaques over her nose and cheeks, along with diffuse facial erythema, papules, and pustules (Figure 1(a)). The father referred chronic use of topical hydrocortisone and betamethasone for over 4 months to treat facial eczematous lesions. The patient was otherwise healthy. A potassium hydroxide (KOH) mount of a pustule scraping revealed several* Demodex folliculorum* mites (Figure 1(b)). Oral ivermectin (200 *μ*g/kg, single dose) plus topical permethrin 5% lotion applied for 3 consecutive nights were administered; afterwards, oral erythromycin 30 mg/kg/day, divided in three doses, plus metronidazole cream was added. Oral ivermectin was repeated every week for a total of 10 doses. Although lesions improved greatly within the following 3 months (Figure 2), oral erythromycin was maintained for 2 months to avoid a recurrence.

## 3. Discussion

Two species of Demodex have been identified in humans:* D. folliculorum*, with a cigar-shaped body usually found within hair follicles, and* D. brevis*, which is smaller and favors the sebaceous glands [3]. While inhabiting the pilosebaceous unit, they feed on sebum and bacteria. Infestation of the pilosebaceous unit by Demodex mites is common, mite density is low in healthy skin, and only few individuals present symptoms [4].

Demodicosis can be classified as primary, in the absence of other inflammatory dermatoses, having a sudden onset, or secondary when associated with other cutaneous or systemic diseases, developing gradually over existing dermatoses [5]. The latter is frequently found in severely immunosuppressed patients, including those using topical corticosteroids or calcineurin inhibitors [6]. Clinical presentation is heterogeneous and can include pruritic papules, vesicles, pustules, plaques, granulomatous, and even cystic facial lesions [5, 7]. Crusted exuberant lesions have already been reported in an adult with HIV infection and chronic use of steroids [8]. A case of demodicosis mimicking favus has been reported in an immunocompetent child [9].

Diagnosis can be made by standardized skin surface biopsy or skin scraping, usually considering abnormal anything more than 5 mites per cm^2^ [6].

There are several treatment options with varied efficacy, although there is a strong lack of evidence-based literature. Ivermectin (200 *μ*g/kg single dose) is the current treatment of choice and can be combined with topical permethrin, benzyl benzoate, or metronidazole [6, 7]. The mechanism of action of antimicrobial agents remains to be fully elucidated, with reports of its efficacy being secondary to their anti-inflammatory effect or by reducing bacteria both living on the mite and that on which the mites feed on [2].

To our knowledge, this is the first reported case of facial crusted rosacea-like demodicosis in a pediatric patient. Usually,* Demodex* colonization is not significant in infants and children due to low sebum production [3].

The pathogenesis and immune response to mite invasion are not clearly understood; thus, the particularly severe clinical manifestations seen in this case could be attributed to local immunosuppression secondary to chronic use of topical steroids. Like the lesions observed in patients with Norwegian scabies, this particular clinical presentation of* D. folliculorum* infestation could be due to other unknown factors besides the local immunosuppression that led to a defective host-defense immune response resulting in a great increase in parasite population.
